# Histone deacetylases: revealing the molecular base of dimorphism in pathogenic fungi

**DOI:** 10.15698/mic2015.11.240

**Published:** 2015-11-04

**Authors:** Alberto Elías-Villalobos, Dominique Helmlinger, José I. Ibeas

**Affiliations:** 1 Centro Andaluz de Biología del Desarrollo (CABD), Universidad Pablo de Olavide -Consejo Superior de Investigaciones Científicas-Junta de Andalucía, ES-41013 Seville, Spain.; 2 Centre de Recherche de Biochimie Macromoléculaire, Centre National de la Recherche Scientifique UMR5237-Université de Montpellier, Montpellier, France.

**Keywords:** histone, acetylation, deacetylation, HAT, HDAC, dimorphism, virulence, pathogenic fungi, Ustilago maydis, Hos2, Clr3, plant pathogen

## Abstract

Fungi, as every living organism, interact with the external world and have to adapt to its fluctuations. For pathogenic fungi, such interaction involves adapting to the hostile environment of their host. Survival depends on the capacity of fungi to detect and respond to external stimuli, which is achieved through a tight and efficient genetic control. Chromatin modifications represent a well-known layer of regulation that controls gene expression in response to environmental signals. However, less is known about the chromatin modifications that are involved in fungal virulence and the specific cues and signalling pathways that target chromatin modifications to specific genes. In a recently published study, our research group identified one such regulatory pathway. We demonstrated that the histone deacetylase (HDAC) Hos2 is involved in yeast-to-hyphal transition (dimorphism) and it is associated with the virulence of the maize pathogen *Ustilago maydis*, the causative agent of smut disease in corn. Hos2 activates mating-type genes by directly binding to their gene bodies. Furthermore, Hos2 acts downstream of the nutrient-sensing cyclic AMP-Protein Kinase A pathway. We also found that another HDAC, Clr3, contributes to this regulation, possibly in cooperation with Hos2. As a whole, our data suggest that there is a direct link between changes in the environment and acetylation of nucleosomes within certain genes. We propose that histone acetylation is critical to the proper timing and induction of transcription of the genes encoding factors that coordinate changes in morphology with pathogenesis.

Plant pathogenic fungi need to undergo a series of morphological changes in particular phases of their infection cycle. The success of infection depends on the proper timing of these changes. *U. maydis* starts its pathogenic cycle with a morphological shift from a yeast-like form to a polarized filament on the surface of the maize leaf, a process controlled by factors encoded by the mating-type loci. The *a* locus encodes for the pheromone-receptor system, which allows the recognition and fusion of sexually-compatible haploid cells. The fate of the resulting dicaryon is then determined by the *b* locus, which encodes for transcription factors that form a compatible heterodimer only when co-expressed by cells of the opposite mating type, thus promoting filamentation and virulence. The expression of genes at the *a* and *b* loci is controlled by the *prf1* transcription factor, which is activated both by the cAMP-PKA pathway and the pheromone-responsive Mitogen Activated Protein Kinase (MAPK) cascade at transcriptional and posttranslational level. The regulation of mating genes is therefore critical to ensuring the initiation of the pathogenic program in the smut fungus. Several transcription factors have been shown to regulate these genes by directly or indirectly affecting *prf1* expression, yet very little is known about their regulation by chromatin-modifying factors. We addressed this question by analysing the role of histone deacetylation in dimorphic switch and virulence in *U. maydis*.

Histone acetylation and deacetylation represent a general and effective way to control gene expression upon changes in the environment. However, little is known about their role during fungal phytopathogenesis. In our work, we generated deletion mutants for all putative class I and II HDACs in *U. maydis*. We found that only the histone deacetylase Hos2 was required to maintain the virulence of the fungus, playing a crucial role in the yeast-to-hypha transition. The deletion of *hos2* affects dimorphic switch in two ways: Firstly, it impairs conjugation tube formation in the two haploid mating partners. Secondly, it reduces filamentation in the resulting dicaryon. The constitutive expression of a compatible *b* heterodimer in a Δ*hos2* strain restored the filamentation capacity of the fungus thus suggesting an under-expression of mating genes in the deletion mutant, which was confirmed by expression analysis. These defects probably explain the reduced virulence of Δ*hos2* mutants. Nevertheless, after the yeast-to-hypha transition, other morphological changes are required for *U. maydis* to penetrate the plant cuticle, expand to plant tissues and produce fungal dispersal elements, i.e. spores. Although we observed that Δ*hos2* cells can produce spores, Hos2 may play other roles in appressorium formation or penetration or during fungal expansion *in planta*.

The analysis of putative redundancies between different HDACs may reveal previously unknown roles of Hos2 and other histone deacetylases. In our work, this was evidenced for the histone deacetylase Clr3, which deletion did not cause any detectable defect neither in mating nor in filamentation, showing only a very slight reduction in its virulence capacity. The double Δ*hos2*Δ*clr3* mutant, however, showed a severe infection defect. Additionally - as we described for Δ*hos2 -* Δ*clr3* cells showed a reduced expression of mating-type genes upon cAMP addition, and this effect was more pronounced in the double Δ*hos2*Δ*clr3* mutant than in single mutants. This phenotype suggests a redundant function of the two HDACs in the control of mating genes. Alternatively, each HDAC could also affect the expression of these genes by independent mechanisms. In our work, we observed that H4K16 acetylation increased in Δ*hos2* cells and that Hos2 bound directly to the gene body of mating-type genes. However, direct targets for Clr3 remain to be defined. Interestingly, in the human pathogen *Candida albicans*, both Hos2 and Hda1 (Clr3 homolog) have been shown to regulate dimorphic switch. Unlike *U. maydis*, Hos2 and Hda1 have opposite roles in yeast-to-hypha transition, because Hos2 inhibits filamentation, whereas Hda1 promotes it. However, under nitrogen starvation conditions, Hos2 is also necessary for the promotion of filament formation, which suggests that, depending on the nutritional conditions, these HDACs can exert either similar or opposite roles in dimorphism. In the light of these findings, the identification of interactions between other histone deacetylases might reveal unknown functions of HDACs in fungal phythopathogenesis.

Histone deacetylation has been traditionally associated with the repression of gene expression; however, there is solid evidence that HDACs can also act as transcriptional activators. This has been clearly demonstrated for Hos2 which, contrary to other HDACs, predominantly binds to gene bodies and contributes to transcriptional elongation by RNA Polymerase II in many organisms. Interestingly, in our work, we detected a significantly higher binding of Hos2 to the promoter of the *prf1* transcription factor, as compared to its gene body. One exciting possibility is the existence of a long non-coding RNA within the *prf1* promoter which regulation depends on Hos2, as previously described for the Hos2 target gene *IME1* in *Saccharomyces cerevisiae*. Significantly, Hos2 binding to the *prf1* promoter - as well as to the body of pheromone and receptor genes - was affected by the addition of cAMP, whereas it was not the case for the *prf1* gene body. Thus, it is possible that Hos2 regulates *prf1* and its target genes by different mechanisms, which would evidence the versatility of chromatin modifiers in the control of gene expression. This long non-coding RNA would represent an additional regulatory mechanism to our current model of how Hos2 regulates mating in *U. maydis* (Figure 1).

**Figure 1:**
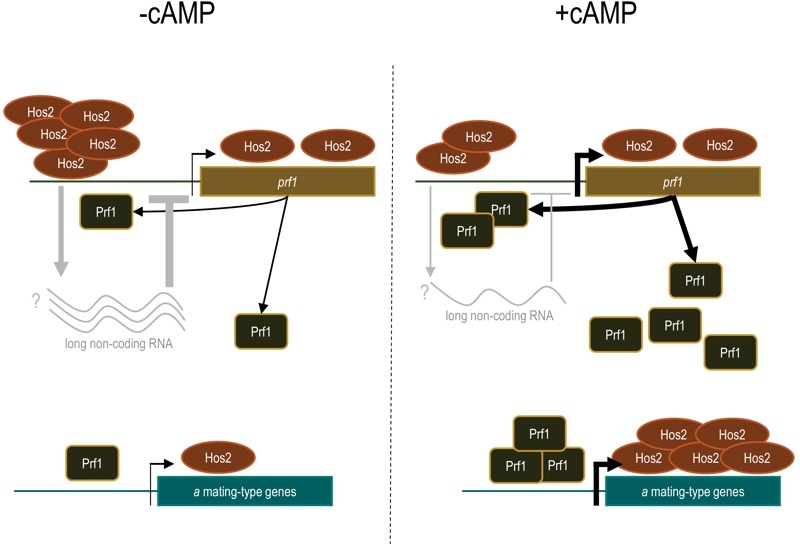
FIGURE 1: Working model for Hos2-dependent regulation of mating. During yeast-like growth and in the absence of cAMP (left), expression of *prf1* and its target genes at the *a* mating-type loci is maintained at a low basal level just to allow cells of opposite mating types to be able to recognize each other. Hos2 binding to the gene bodies of mating genes might help to maintain this basal expression. During mating or after the addition of cAMP (right), the expression of *prf1* and *a* genes increases significantly. Concomitantly, Hos2 binding to the gene bodies of *a* genes increases too, probably contributing to their transcriptional elongation and thus allowing efficient mating. Though Hos2 binding to the *prf1* gene body remains constant, it decreases at the UAS of its promoter. A plausible explanation for these results is the existence of a long non-coding RNA that inhibits *prf1* expression during yeast-like growth (grey lines and arrows).

The fact that histone deacetylases play an important role in dimorphism and virulence suggests that opposite enzymatic activities, histone acetyltransferases (HATs), are also involved in the regulation of these processes. Indeed, *U. maydis* Gcn5 HAT has recently been proven to control dimorphic switch. Interestingly, the deletion of Gcn5 leads to a constitutive filamentation phenotype, thus opposing the effect of *hos2* deletion. It is tempting to speculate that both histone modifiers are responsible for the coordination between yeast and filamentous growth through acetylation or deacetylation of histones at the same target genes. A third factor may contribute to the control of the dimorphic switch and help to understand this relationship. In our study, we demonstrate that Hos2 and the general transcriptional repressor Tup1 play independent roles in the control of virulence in *U. maydis*. Hos2 acts downstream of the cAMP pathway, whereas Tup1 acts within the MAPK cascade (as shown in a previous study). Given that in *S. cerevisiae* Tup1 prevents Gcn5 recruitment to gene promoters, we hypothesize that Gcn5 and Tup1 would act together in the control of gene expression and dimorphism, whereas Hos2 would act in a parallel pathway. However the signalling pathways through which Gcn5 controls dimorphism remain to be elucidated. The generation of double mutants will be necessary for studying functional interactions between these factors in the control of dimorphism and virulence.

Each chromatin modification should not be examined as an isolated event regulating gene expression, but they are rather integrated into a more complex network of regulatory steps. Apart from redundancies among chromatin modifiers, there are extensive cross-talks between distinct modifications within chromatin, thus increasing the versatility of gene regulation and allowing cells to fine-tune their response to changes in their environment.

In summary, our work provides a new insight into the response mechanisms of chromatin modifiers to external stimuli, contributing to a better understanding of the genetic regulation of dimorphism in pathogenic fungi and of how environmental signals regulate gene expression.

